# Continuous Three-Dimensional Control of a Virtual Helicopter Using a Motor Imagery Based Brain-Computer Interface

**DOI:** 10.1371/journal.pone.0026322

**Published:** 2011-10-26

**Authors:** Alexander J. Doud, John P. Lucas, Marc T. Pisansky, Bin He

**Affiliations:** Department of Biomedical Engineering, University of Minnesota, Minneapolis, Minnesota, United States of America; The University of Western Ontario, Canada

## Abstract

Brain-computer interfaces (BCIs) allow a user to interact with a computer system using thought. However, only recently have devices capable of providing sophisticated multi-dimensional control been achieved non-invasively. A major goal for non-invasive BCI systems has been to provide continuous, intuitive, and accurate control, while retaining a high level of user autonomy. By employing electroencephalography (EEG) to record and decode sensorimotor rhythms (SMRs) induced from motor imaginations, a consistent, user-specific control signal may be characterized. Utilizing a novel method of interactive and continuous control, we trained three normal subjects to modulate their SMRs to achieve three-dimensional movement of a virtual helicopter that is fast, accurate, and continuous. In this system, the virtual helicopter's forward-backward translation and elevation controls were actuated through the modulation of sensorimotor rhythms that were converted to forces applied to the virtual helicopter at every simulation time step, and the helicopter's angle of left or right rotation was linearly mapped, with higher resolution, from sensorimotor rhythms associated with other motor imaginations. These different resolutions of control allow for interplay between general intent actuation and fine control as is seen in the gross and fine movements of the arm and hand. Subjects controlled the helicopter with the goal of flying through rings (targets) randomly positioned and oriented in a three-dimensional space. The subjects flew through rings continuously, acquiring as many as 11 consecutive rings within a five-minute period. In total, the study group successfully acquired over 85% of presented targets. These results affirm the effective, three-dimensional control of our motor imagery based BCI system, and suggest its potential applications in biological navigation, neuroprosthetics, and other applications.

## Introduction

**Figure 1:**
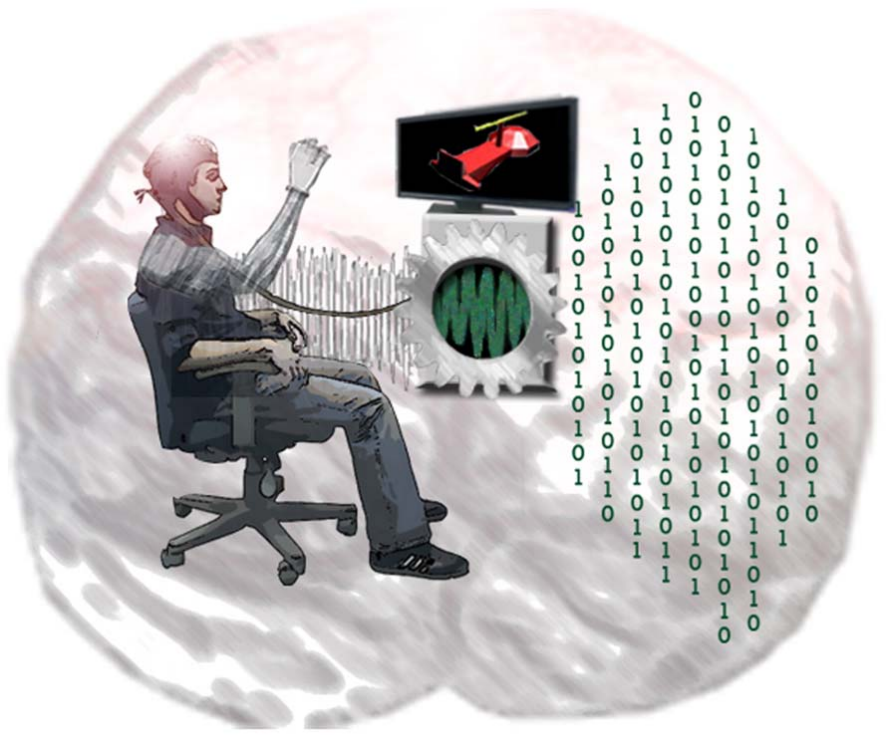
A diagrammatic representation of the presented BCI system. Using specifically trained motor imaginations learned in single dimensional cursor tasks, subjects control the three-dimensional movement of a virtual helicopter. Raw EEG is temporally and spatially filtered to produce individualized control signal components. These components are weighted and digitized in a subject specific manner and output to influence control in the virtual world.

A brain-computer interface (BCI) is a system that interprets the thoughts of the user to produce commands that control a computer or device. Many systems adapt to the user, who in turn is continually adapting to the system. The role of this feedback-adaptation loop between the system and user is of considerable importance in BCI systems that attempt to approximate neural function (Figure 1).

Until recent years, sophisticated thought-based control of movement in multiple dimensions was relegated to the subcategory of invasive BCI systems [1], [2], [3], [4], [5], [6], [7]. While these invasive BCI systems have shown great promise for controlling an external device from signals extracted from the brain of animals or human subjects, these systems present various degrees of risk associated with the implantation of a recording device in the subject's brain. As such, they are practicable only in cases where motor ability is extremely impaired and alternate communication methods are infeasible.

Parallel to invasive BCIs, noninvasive BCI systems using EEG or other signals have been pursued [8], [9], [10], [11], [12]. In recent years, advances have been made in acquisition, filtering, and data processing capabilities that allow for an approximation of the abilities of the invasive systems using non-invasive EEG [13], [14]. Such systems have provided users the ability to explore virtual environments [15], [16], enter text in typing programs [17], control interactive robotic wheelchairs [18].

The recording and classification of sensorimotor rhythms (SMRs) associated with trained motor imaginations have heretofore given users control of a computer cursor in two dimensions [19], [20], and recently up to three dimensions [21], [22]. Methods for training users in single-dimensional cursor control are well established and the use of a control signal generated from SMRs has been well characterized [23], [24], [25]. By training single dimensions of control independently, subjects can progress incrementally to master cursor control in 2D space [20]. However, making the transition between 2D and 3D control remains difficult. Users must simultaneously orchestrate the production and adjustment of multiple, often unrelated mental tasks to produce independent control signals. Many applications address this challenge by presenting limitations in the form of requirements for fixed, discontinuous control intervals, *a priori* assumptions about user intent and constrained control spaces [18], [21]. Here we have employed novel control strategies to give the subject both gross and high-resolution control. In so doing, the proposed system may overcome the necessity for these constraints.

We previously demonstrated a reductionist approach to the 3D control problem in which subjects used two-dimensional control to gain mastery of 3D space [22]. Subjects controlled the altitude and rotation of a virtual helicopter that moved with a constant forward velocity. This quasi-3D helicopter simulation served well as a transitional task to ease subjects into true control of three-dimensional movement. Here we have added a third control signal to modulate the helicopter's forward and backward movement and present a system that gives users continuous control of the virtual helicopter in three-dimensions. Furthermore, we have refined the control task in order to train subjects in task relevant control, and present expanded EEG analysis during continuous 3D helicopter control. By producing a unique set of motor imaginations and a volitional “rest” state to control vertical, rotational, and forward-backward motion, users learned to fly the helicopter quickly, accurately, and continuously through a series of rings presented randomly throughout the virtual 3D space.

## Methods

### Study Overview

The study consisted of two experimental controls and the virtual helicopter control task. Prior to engaging in the helicopter control task, each subject was given the opportunity to become familiar with the control environment and to adjust control parameters to suit each subject's preference. During this pre-task phase, subjects were encouraged to explore the environment and were given the opportunity to adjust the strength of actuation of each direction of movement according to personal preference. During the helicopter control phase, subjects were instructed to attempt to fly through as many rings as possible in each five-minute trial. They were instructed to avoid colliding with the ring, the edges of the control space and virtual buildings. Between each trial, subjects were given the option to adjust control parameters according to preference.

### Experimental Subjects

Three healthy human subjects participated in this study (all female, aged 20–23 years), who all gave written consent according to a protocol approved by the Institutional Review Board of the University of Minnesota. Two of the subjects (Subjects 1 and 2) had previously learned to control the quasi-3D virtual helicopter [22].

### Subject Training

All subjects underwent a sequential training protocol that began with a 1D cursor task utilizing left/right arm, legs, tongue, and rest imaginations. It is important to note here that in this system, volitional “rest” was tied to a direction of control and not an absence of movement. Comparing a subject's neural signal when intentionally resting to the signal produced when imagining both hands produced a highly characteristic control signal. BCI2000's Offline Analysis toolkit was used to determine this and other highly statistically separable (high R^2^ value) locations and frequencies associated with imagination pairs performed during the 1D tasks. Customized electrode and frequency selections for each subject were continuously updated throughout the training process in order to optimize performance. Final electrode and frequency selections are presented in Table S1. Only after proficiency with each motor imagination – as judged by >80% target hit rate – were subjects allowed to progress to the 2D cursor task, which acted to combine select aforementioned imaginations (e.g., left/right arm and legs/tongue). The final training stage introduced subjects to the pseudo-3D helicopter task, as detailed by Royer et al., 2010 [22]. During this stage, subjects familiarized themselves with the virtual environment and helicopter flight. Prior to the experimental sessions presented here, Subjects 1, 2, and 3 completed 4, 11, and 3 sessions (8–9 five-minute runs per session), respectively, in which they were exposed to the new 3D virtual helicopter protocol and the system was adjusted to produce subject-specific and optimized control signals. In these sessions, subjects focused on learning to control the system, and were not tasked with flying through rings.

### Data Collection

Participants sat in a comfortable chair facing a computer monitor. A 64-channel EEG cap was securely fitted to the head of each subject with cap placement consistent across sessions. EEG signals were filtered from DC – 200 Hz and sampled at 1000 Hz by a Neuroscan amplifier (Synamps 2) and imported to BCI2000 [17] without spatial filtering. Subjects performed 5 consecutive experimental sessions (8–10 five-minute runs per session). During these sessions, subjects were visually monitored for inappropriate use of eye or muscle movement. In this subject population, blinking and movement was very minimal. Blinking and small movements that did occur were infrequent, and so presented neither an appreciable impediment nor assistance to the control task. The spectral amplitude of the EEG waveform at a set of specific electrode locations and frequency bins (3 Hz width) was integrated by BCI2000 to produce a control signal that was sent every 30 ms via UDP port to the Blender game environment.

### Virtual Environment

The virtual environment was modelled from the Northrop Mall area of the University of Minnesota in Minneapolis. Subjects were instructed to navigate the helicopter through individually presented and randomly oriented rings, while avoiding collisions with buildings and ring borders. The volume of the helicopter's flight zone measured in cubic Blender units (bu^3^), a native unit of volume in the virtual game, was 4,285 bu^3^. In comparison, the helicopter was small, measuring only 0.24 bu in length along its longest edge. Ring presentation occurred within a centrally-fixed 69 bu^3^ space. Buildings were presented to provide spatial reference and also served as minor obstacles. The buildings occupied roughly 5% of the total environment. Thus, the virtual world did not restrict the helicopter to the area in which targets were presented.

**Figure 2:**
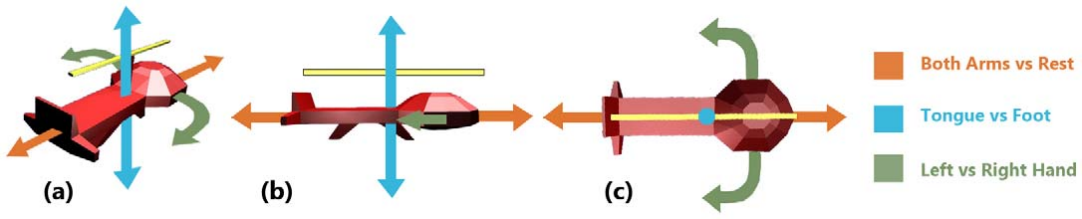
Three-dimensional helicopter control arrangements are shown in perspective (a), side (b) and top (c) views. Users have independent control of forward, backward, up, down, and left and right rotation about the helicopter's z-axis. To go forward or back, subjects imagine moving or resting both hands respectively. To rotate the helicopter left or right, subjects imagine moving either the left or right hand respectively. Subjects imagine moving the tongue to raise the helicopter and moving the feet to lower it. Each control can be independently adjusted in strength according to user preference.

Subjects used three control signals to steer the helicopter. The imaginations associated with each of the helicopter's controls are presented in Figure 2. Subjects imagined moving both hands to move the helicopter forward and imagined no movement to go backwards. They imagined moving right or left hands to rotate right or left, respectively. To raise or lower the helicopter, subjects imagined moving the tongue or feet, respectively. By alternating between imaginations, subjects could maintain a position or orientation, however the majority of trial time was spent pursuing rings or orienting the helicopter to prepare for pursuit. A four point moving average was applied to the LR (left-right) signal to smooth the control. This control was linearly tied to the helicopter's rotational actuator as described in [22]. The FB (forward-backward) and UD (up-down) signals were cubed and translated to forces applied by the simulation's physics engine on the helicopter along each control signal's appropriate axis. The strength of control in each of the six directions of motion could be adjusted between trials by changing linear scale factors according to subject preference.

### Experimental Paradigm

**Figure 3:**
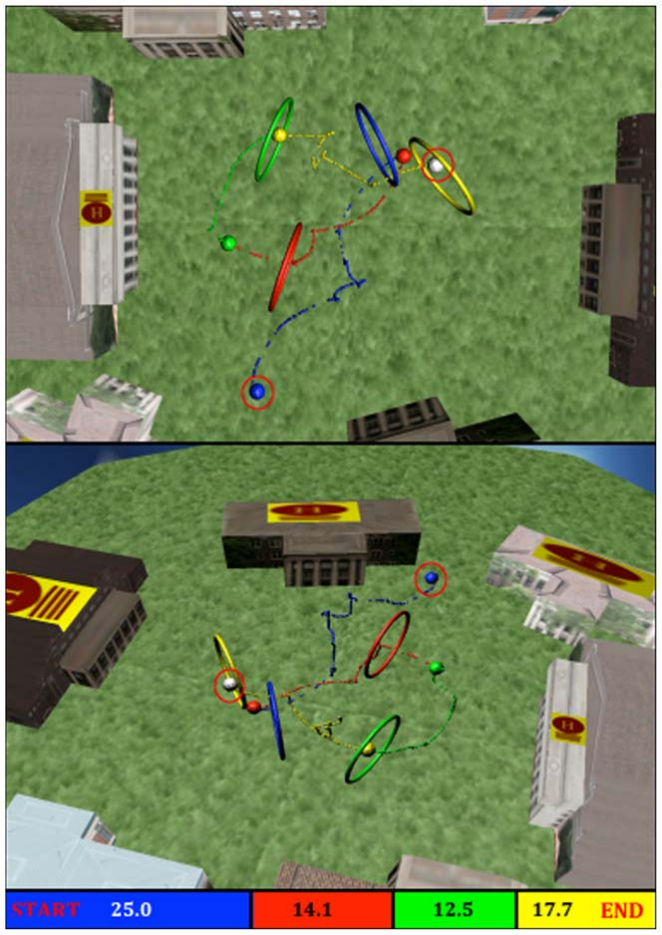
Speed, accuracy, and continuity of control are depicted in a characteristic run performed by Subject 1 (Session 5, Trial 9, Targets 4–7). Only one target was visible to the subject at any given time. The presentation of a new ring occurred 1.5 seconds after a ring hit. The helicopter's position upon ring presentation is represented by a larger coloured sphere. Smaller coloured spheres represent the position of the helicopter sampled every 30 milliseconds. The subject started with the blue ring, and progressed through red, green, and yellow rings as illustrated by the colour bar on the bottom of the figure. The overall duration of this continuous portion of the run was 69.53 seconds.

Subjects completed 5 consecutive experimental sessions over no longer than a three-week period. Each session consisted of 8–10 five-minute runs (for a total of 44, 44, and 45 runs for subjects 1, 2, and 3 respectively). The helicopter started each run on the ground, centred within the target domain. During the first 3 seconds of each run, the helicopter remained stationary before subjects gained control of its motion. Randomly oriented rings were individually presented throughout the run. Subjects were asked to fly the virtual helicopter through as many rings as possible without colliding with a building, the edge of a ring, or leaving the boundaries of the virtual environment. If the helicopter passed through the centre of a ring, one ‘hit’ was recorded and a new target was immediately presented. A representative series of successful hits is shown in Figure 3. Alternatively, if the helicopter collided with a building or reached the edge of the environment, one ‘miss’ was recorded. After a miss, the helicopter was reset to its initial position and a new ring was presented. An ‘invalid’ was recorded if the helicopter touched the edge of a ring. This outcome resulted in the helicopter being reset to the starting position and a new ring being presented.

Subjects were presented with a third person view facing forward from behind the helicopter. A dashboard was located at the bottom of the subjects' monitor, which included a miniature map that depicted the location of the current ring. The dashboard also reported the number of hits obtained and time remaining in the run. Additionally, a virtual joystick was centred on the dashboard. Its vertical position corresponded to the integrated FB control signal and its horizontal position to the integrated LR control signal.

### Experimental Control

Two control experiments were devised to allow for comparison of subject performance. The first experiment aimed to quantify the performance of random noise BCI control. To this end, two subjects who were naive to motor imagery tasks were exposed to the same experimental setup as described previously. Each subject was instructed to sit motionless and maintain a fixed gaze upon the centre of the monitor, while watching video of artificial helicopter movement (i.e., controlled via keyboard by an investigator). Simultaneously, the subjects' EEG rhythms were processed using default control signals that controlled the motion of an unseen, separate helicopter simulation. Subjects were informed that they had no control over the helicopter in the video that they were observing. Additionally, they received no feedback regarding the second helicopter simulation that was simultaneously being controlled via the EEG control signal. Metrics of performance were assessed for the simulation which was under the control of the subject's brain signals but which was not being visually attended to. In this way, control subjects were exposed to visual stimuli similar to that of the experimental subjects but which had no information relevant to the simulation under subject control. In this way, the unattended simulation performance could be attributed to fluctuations in the default control signals caused by natural physiological variability arising from each subject's passive observation of the game stimuli without causal feedback (i.e., without intent to control the system). This control helps to characterize the inherent difficulty of the task and the likelihood for successful task completion in the absence of subject intent.

The second experiment was carried out to evaluate performance when given ideal control of the helicopter. This was accomplished using keyboard controls, which controlled the helicopter in an analogous manner to that of the BCI: rotation was controlled by left/right arrows, vertical displacement was controlled by up/down arrows, and forward/backward displacement was controlled by space/”b” keys. The velocities or rotations assigned to each direction of the keyboard control were the averaged displacement or rotation per frame from the three experimental subjects. The effectiveness of keyboard control was assessed as the number of rings obtained by a subject with no prior exposure to the virtual environment.

### Performance Analysis

Experimental performance was assessed by calculating metrics that reflected the degree of accuracy, speed, and continuity of control. These measures were percent accuracy, average ring acquisition velocity (ARAV), and average rings obtained per reset (ARR). Percent accuracy was determined for two different criteria. Firstly, percent valid correct (PVC) accuracy was calculated by determining the fraction of hits to valid outcomes. Thus, invalid outcomes corresponding to ring collisions were not included in the calculation. Alternatively, percent total correct (PTC) accuracy was calculated by dividing the number of hits by the number of total outcomes (including invalids). In this case, invalids and misses were penalized equally. ARAV, a measure of system speed, was calculated by dividing the absolute distance travelled to acquire a target by the time from target presentation to acquisition. The units of ARAV were helicopter-lengths (0.24 bu) per second. Finally, ARR was used to determine the continuity of control, and was calculated by averaging the number of rings acquired prior to each helicopter reset.

### *EEG Data Analysis*

Single-arm motor imagery has been shown to evoke contralateral event related desynchronization (ERD) and ipsilateral event related synchronization (ERS) in the motor cortex [26], [27], [28]. Thus, a control signal may be characterized by the difference in synchrony between the hemispheres. Here, this idea is utilized in each subject's LR control signal. The mu rhythm spectral amplitude is negatively weighted at electrodes located in the left hemisphere and positively weighted at electrodes in the right hemisphere. Therefore, the summation of these components during right-arm imaginations yields positive values and during left-arm imaginations yields negative values.

To this end, the changes in spectral amplitude of 3 Hz, subject-specific frequency bins were extracted from the raw EEG using BCI2000. The normalized contributions of the left side electrodes were differentially weighted and subtracted from the weighted contribution of the right electrodes. The result was normalized continuously to produce a control signal of zero mean and unit variance. Because of normalization, relative low amplitude in an electrode corresponded to a negative valued contribution from the electrode. In the case of a right turn, the subject imagined the use of the right hand to produce reduction in amplitude of the mu band of the left electrode and increased amplitude in the mu band of the right electrode. This corresponded to a positive contribution from the right electrode and a negative contribution from the left electrode. Since the left electrode was subtracted from the right, the result was a strong positive control signal that incorporates both the ERD and ERS events. For a left turn, the same logic applies and results in a strong negative control signal. This signal was then received by the helicopter simulation and processed as described in equation 1 and applied as an angle of rotational displacement.
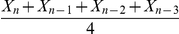


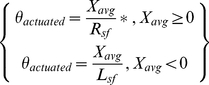
(1)The left-right actuation of the helicopter's movement was a 4-point moving average calculated from the signal coming to the game every 30 ms. The averaged signal was then scaled (for positive values with a right scale factor, 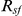 and for negative values with a left scale factor, 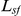). These scale factors converted the signal to a displacement in radians that updated the left or right rotation of the helicopter in the simulation at every time step.

Translational control of forward-backward movement and elevation was actuated as described in equation 2 and applied to the helicopter as forces at each time step.
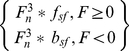
(2)To produce changes in the forward-backward translation of the helicopter the forward control signal was translated to an actuated force by multiplying the positive cubed signal values by a forward scale factor, 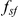 and the negative cubed signal values by a backward scale factor, 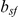. Scale factors were chosen based on experience with the game's physics engine, and were refined according to subject preference reported during training runs. An analogous approach was used for actuation of the helicopter's elevation control.

Through this control approach, a strong signal actuated as a force could have a lasting effect on the helicopter's forward-backward translation or elevation, but would contribute for only a short time if actuated as a turn. In this sense, the subjects had control of both high-resolution angle actuation for turning the helicopter and lower resolution force actuation to control forward-backward movement and elevation.

To produce forward and backward movements, the same hand movements were used in a different proportional arrangement. For forward and back, the left and right control electrodes were added. Accordingly, the subject was instructed to imagine the use of both hands to go forward and to rest to go back. The expectation was that the imagination of both hands would produce approximately equal desynchronization in the left and right control electrodes. This would produce a negative contribution from both hemispheres that when added would result in a strong negative control signal. The rest state would desynchronize both electrodes approximately equally and would produce, by the same argument, a strong positive control signal. When both hands or rest were imagined, the magnitude and sign of the contribution of each contributory electrode should theoretically be approximately equal. This would produce a value close to zero for the left-right control signal, since it resulted from the subtraction of the two sides as described above. This approach of using different pairings of hand movements to allow for separable control states is an established method for producing a 2D control signal [20].

**Figure 4:**
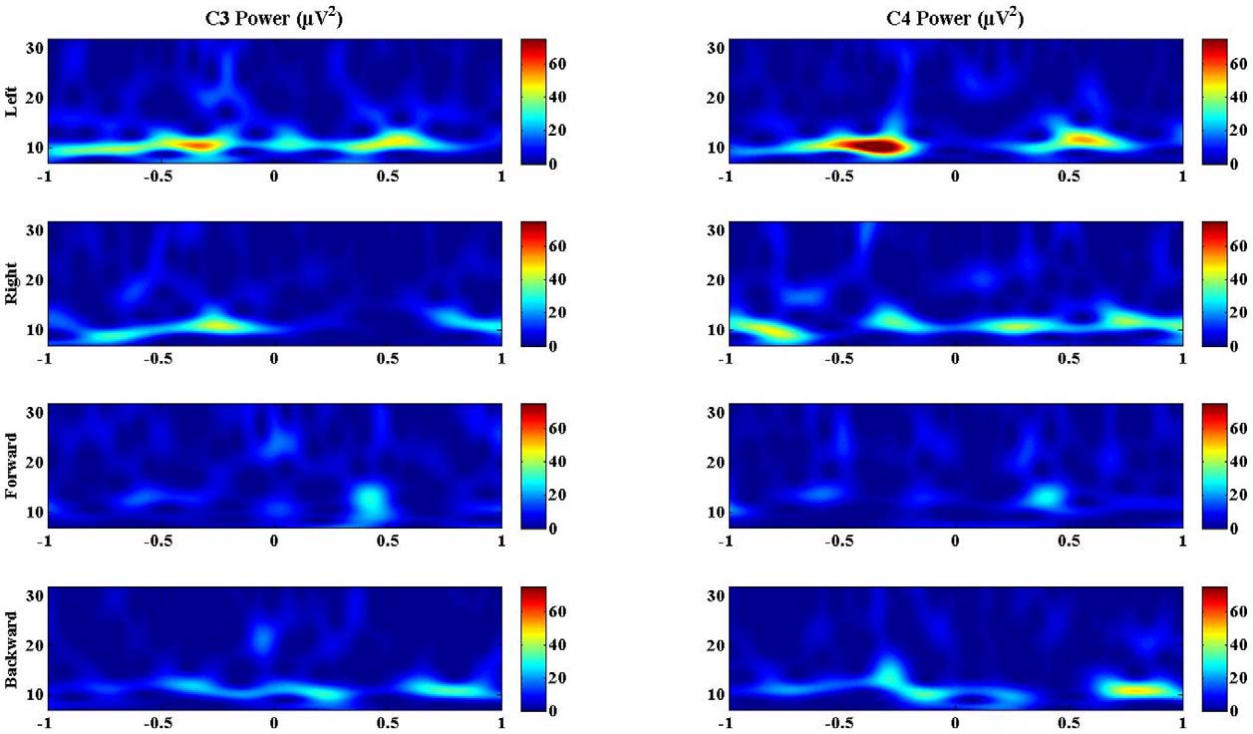
Time-frequency analysis shows averaged power distributions across time and frequency for representative control electrodes during segments of single direction control for subject number 3. Electrode C3 is on the left scalp hemisphere and electrode C4 is on the right. At time 0, the subject moved the helicopter in primarily one direction for .5 s. When a right turn is made, C3 shows ERD and C4 shows ERS. The opposite is true for a left turn. When both hands are imagined, both electrodes show periods of desynchronization, while the rest state results in both electrodes exhibiting synchronization. These changes in the time-frequency profiles may be leveraged to control two-dimensions of movement with only hand imaginations and volitional rest.

Figure 4 demonstrates this control arrangement by examining the EEG data associated with a single direction of movement of the helicopter during which other dimensional movements were minimal. The control signal was analyzed to select segments of EEG in which the helicopter was performing primarily a single direction of movement for 0.5 sec (right, left, forward, or backward) and concurrent movement in other directions was minimal. Time-frequency analysis shows the distribution of power associated with each imaginative state during online virtual helicopter control. The figure shows that the helicopter's turns resulted from the ERD of the electrodes contralateral to a given motor imagination and ERS of the ipsilateral electrodes. Forward and back movement resulted from increases in power in both electrodes associated with the rest imagination state and decreases in power associated with the imagination of both hands. Thus, the separation of four control states in the context of the continuous online helicopter control task was made possible through the use of differential modulation of hand imaginations.

In practice, the correlation between control signals varied greatly between subjects and within the same subject across trials. Since the forward-back and left-right control signals both relied on modulation of hand imaginations, it is reasonable to expect that the correlation between these control signals was elevated when compared to the other correlation pairs. Figure 4 demonstrates that while the correlation between subjects left-right and forward-back control signals was greater than that of other dimensional pairings, the correlation covers a wide range across the subject population and across trials within the same subject. This implies that the degree of dependence of the two hand based dimensional controls is dynamic. Further investigation of how this coupling changes during a single run or even a single trial may be an interesting direction for future investigation.

## Results

### Subject Accuracy of Control

**Figure 5:**
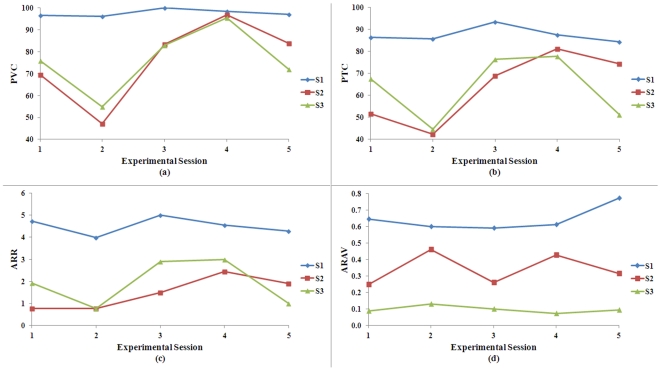
Performance quality metrics. (a) Percent valid correct (PVC) is the ratio of total hits to total non-invalid attempts during each experimental session. (b) Percent total correct (PTC) includes invalid attempts in the calculation. (c) The average number of rings obtained per reset (ARR) is a metric of control continuity. (d) The average ring acquisition velocity (ARAV) is the average of the net distance travelled by the helicopter from ring presentation to acquisition divided by the time required to cover the distance. ARAV serves as a control speed metric.

The experimental subjects achieved accurate control of the virtual helicopter in three dimensions. This is presented in Figure 5 (a, b) as the percent accuracy of control over the five consecutive experimental sessions. The figure reports the average percentage of presented targets that the subjects successfully passed through in a given experimental session, and is shown for both PVC and PTC scoring criteria. It should be noted that while PTC is a pragmatic assessment when considering control of a real helicopter, it does not adequately reflect the degree of subject control required to fly the helicopter to touch the target, since a small error at the end of a nearly perfect flight results in a invalid attempt if the helicopter contacts the ring's edge. The PVC is a metric, which does not include invalid trials in the number of attempts, and so neither rewards nor penalizes collisions with the ring. Many BCI studies only require the subject to contact the target with the controlled cursor [17], [18], [20], [21], [25]. By comparison, both of the reported metrics we employed in the present study are stricter measures of control accuracy, as success requires the subject to both plan the path taken such that the ring is correctly oriented and to avoid the ring edges while passing through it.

Subjects achieved high accuracy under both metrics, with Subject 1 achieving an average PVC accuracy of 97.6% and an average PTC accuracy of 87.31% (of 331 attempts). Subject 2 achieved an average PVC accuracy of 72.2% and an average PTC accuracy of 61.3% (of 191 attempts). Lastly, Subject 3 achieved an average PVC accuracy of 76.5% and an average PTC accuracy of 63.3% (of 180 attempts). Subject 1 showed consistent performance throughout the 5 consecutive experimental sessions with a maximum session PVC and PTC accuracies of 100% and 93.3%, respectively. Subject 2 showed steady improvement over the course of the 5 session series and achieved a maximum session PVC and PTC of 96.8% and 81.1%, respectively. Similarly, Subject 3 exhibited an improvement in scores over sessions, with a maximum PVC and PTC of 95.5% and 77.8%, respectively. The difference between PVC and PTC scores indicates the contribution of ring collisions to subject error. In these invalidated trials, subjects often had excellent control of the helicopter, but happened to contact the edge of the ring while attempting to pass through. As stated previously, penalization of this type is not implemented in other multidimensional BCI studies, but was intentionally chosen here to motivate subjects to achieve the finest degree of control of the virtual helicopter.

### Experimental Control Results

For the first control scenario employing random noise, each of the two subjects completed 9 5-minute sessions (total of 90 minutes helicopter flight time), having 55 and 32 respective attempts at targets. Only one ring hit was recorded during the entire control experiment. The control subjects acquired 1.15% of 87 targets, while the experimental subjects acquired 73.69% of 707 targets. Thus, the experimental group showed accuracy scores roughly 64 times greater than the control group, making statistical comparison inessential.

For the second control scenario aimed to assess optimal acquisition rate, each of the two control subjects completed 15 5-minute sessions (total of 150 minutes helicopter flight time) prior to which they were instructed to obtain as many rings as possible and to avoid collisions with extraneous objects in the environment. Under these conditions, the subjects were able to acquire 100% of presented rings with an average of 31 and 29 respective rings acquired per session. Comparatively, Subjects 1, 2, and 3 acquired 87.3%, 61.3%, and 62.2% of presented rings with an average of 6.57, 2.66, and 2.50 respective rings acquired per session. Thus, keyboard control demonstrated largely more efficacious ring acquisition than EEG-based control.

### Subject Speed of Control

Subjects learned to steer the helicopter rapidly through three-dimensional space by modulating control of the FB control signal. This was the major factor that increased the maximum speed of this system over our past work with the virtual helicopter. In the previous work [22], subjects relied on a constant forward velocity to remove the necessity for a third control signal. Here, subjects reported using their LR and UD control signals to make fine adjustment to align the helicopter with the ring before accelerating forward through the target. This strategy can be observed in the supplementary videos. In videos S1 and S2, subjects dampen forward motion during the fine adjustment phase, and then accelerate forward when properly aligned with the target. This deliberate planning allowed for control before, during, and after target acquisition and was an important part of achieving fast, accurate, and continuous control.

While subjects were capable of moving backwards, this functionality was mainly used as a brake to avoid collision and not as a major translational control. This was likely due, in part, to the fact that subjects could not see their path when moving backwards. Video S3 shows trials in which Subject 2 was asked to use backward control to acquire a ring. It is clearly seen that Subject 2 was able to pass through the ring using backward movement. All three subjects demonstrated the ability to acquire rings by moving backward.

The speed imparted to the system through control functionality is summarized in Figure 5d. The figure presents calculated ARAV over each of the 5 consecutive experimental sessions. ARAV is a metric that allows for the characterization of the speed of pursuit of randomly positioned targets presented sequentially. For the five consecutive experimental sessions, Subject 1 recorded an ARAV of 0.65 helicopter lengths per second, Subject 2 recorded an ARAV of 0.36 helicopter lengths per second, and Subject 3 recorded an ARAV of 0.10 helicopter lengths per second. To give the reader a sense of real-world equivalent values, if the helicopter's length were scaled to the standard length of an AH-64 Apache military helicopter, Subjects 1, 2, and 3 would effectively be pursuing the presented targets at an average velocity of 25.5 mph, 14.0 mph, and 3.82 mph, respectively.

### Continuity of Control

Subjects controlled the virtual helicopter in a continuous path through space. When a subject passed through a ring, a new ring was presented. Thus, a characteristic flight path consisted of passing through several consecutive rings without being reset to the starting point. Figure 5c shows the average number of consecutive ring hits prior to a reset (ARR) for each of the 5 consecutive experimental sessions. Subjects 1, 2, and 3 acquired ARR scores of 4.5, 1.5, and 2.0, respectively, over the 5 consecutive experimental sessions. Subject 1 set a study high score of 11 consecutive rings in a 5-minute period. Subject 1 averaged 4 or more consecutive rings for each of the five experimental sessions, while Subject 2 and 3 gained proficiency in continuous control over the course of the sessions. Video S1 shows Subject 1 flying through three consecutive rings within a 30 second period. Video S2 shows subject 2 passing through two rings within a 30 second period. Similarly, video S4 shows subject 3 passing through 3 rings within a 40-second period. These supplementary videos demonstrate the subjects' continuous control over the three dimensions of rotation, elevation, and forward and backward movement.

### Exclusivity of Control Signals

**Figure 6:**
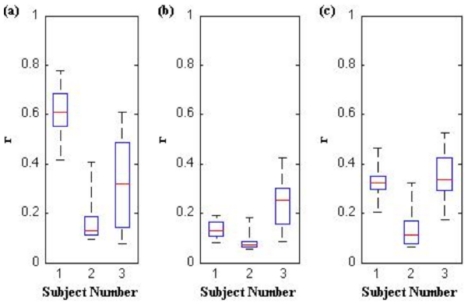
Correlation coefficients between (a) LR and FB (b) LR and UD, and (c) FB and UD control signals at zero lag. Calculations were made for each five-minute session.

To assess the degree to which subjects use control signals independently, each control signal pairing (i.e. left/right and forward/backward) was cross-correlated at zero time lag across each five-minute session. Figure 6 shows the results of these calculations. With the exception of Subject 1's LR and FB control pairing, all median correlation coefficients were less than 0.37 for each signal pairing. This suggests that control signals were predominately independent. One possible explanation for the high correlation between Subject 1's LR and FB control signals is her preference to use forward and rightward movements simultaneously. When questioned about her ability to separate these controls, she could move forward or turn right independently. The ability to modulate both the LR and FB control signals simultaneously was perhaps advantageous, as evidenced by her exemplary performance compared with Subjects 2 and 3.

## Discussion

The introduction of a third control dimension allowed for successful navigation of three-dimensional space that was fast, accurate, and continuous. Two of the experimental subjects (Subject 1 and 2) presented in this work had trained previously with the virtual helicopter using a modified reductionist control strategy that allowed for three-dimensional control by using only two control signals [22]. It is important to note that the differences in subject training prior to participation in this study preclude an in depth examination of the absolute difficulty subjects faced when learning the task. However, observations of subject experience are qualitatively informative. The transition to three-dimensional control appears to have been straightforward for Subject 1, as evidenced by a consistently high level of performance over the course of the five consecutive experimental sessions. Subjects 2 and 3 adjusted to the new control paradigm more gradually; both gained proficiency over the course of the experimental sessions. With the presented work proving the efficacy of the proposed system in the 3D control task, a more rigorous treatment of learning in multidimensional virtual helicopter control may be an informative direction for future efforts.

Unlike Subject 1 and 2, Subject 3 had no previous experience using the modified reductionist control strategy to control the virtual helicopter. Rather, this subject completed only 1- and 2-D cursor task training prior to her involvement with 3D virtual helicopter task. Despite her relative inexperience, this subject demonstrated higher PVC/PTC and ARR scores than Subject 2, a more extensively trained subject. This lends credence to a less extensive training regimen for future subjects prior to introduction to the 3D virtual helicopter task. Moreover, Subject 3 also exhibited improved performance over the course of the 5 sessions, leading us to believe that further improvement in performance could be achieved with additional experience in the helicopter task.

Subjects were trained to accurately fly the helicopter through three-dimensional space. An important part of this training was the requirement that they pass through the ring without hitting its edges. The PVC and PTC accuracy assessments presented in Figure 5 take this additional requirement into account and are stricter measures of control accuracy than those reported in conventional BCI cursor tasks. By imposing these conditions, subjects must not only reach the target space, but also plan and execute an appropriate flight path that avoids the ring's edge. This action often necessitated the simultaneous orchestration of multiple control states, continuous adaptation to system feedback and modulation of the strength of imaginations. Subject 1 in particular described how, over the course of training, this process transitioned from the use of definitive imaginative tasks to the ability to shift awareness to the arms, legs, or tongue. Thus, a subject's motor imagery abilities may evolve from representational imaginations to more abstract and intuitive control over the course of training.

Subjects used the FB control signal to rapidly fly through rings after properly aligning the helicopter. This is seen in several of the supplementary videos and reflects the general strategy for ring acquisition. Between individual trials, subjects were able to request adjustment in the relative strength of each of the control components. Recurring subject selections resulted in an optimization of directional velocities, including a general attenuation of the backward control strength. However, the backward control remained a viable option for breaking or backing away to avoid obstacles. This is probably because the third person view presented to the subject was linked to the helicopter's motion and was oriented forward. Therefore, subjects could not see obstacles or rings that were behind them when moving backward. This strategy is not uncommon in the real world. Real helicopters and cars use slow backward motion for adjustment or obstacle avoidance even though more rapid backward motion is possible. When subjects wanted to go quickly in the opposite direction, they preferred to rotate the helicopter 180 degrees and then use the forward control. Yet, when asked to do so, all subjects were capable of flying through rings in reverse. By optimizing the control signal, the independent control component weighting, and the strategy employed, subjects were able to pursue rings quickly through 3D space. This speed of control is reflected in the ARAV values reported in Figure 5d.

Continuity of control was an important objective of this study. To be considered continuous, control must allow for the acquisition of greater than one target in an unbroken control path. Continuous control was achieved by presenting subjects with a series of randomly oriented targets throughout a 3D environment. Figure 5c reflects the degree of continuous control achieved by each subject. All subjects averaged more than 1 ring prior to a reset for the 5 consecutive experimental sessions (4.5, 1.5, and 2.0 for Subjects 1, 2, and 3 respectively).

The experimental protocol was designed to reward the development of control that was fast, accurate and continuous. By requiring that subjects fly from one ring to the next, subjects learned to modulate their control before, during and after ring acquisition. Significant time penalties were associated with resetting after collisions with objects. Thus, intentionally colliding with an object to be reset within the target domain when presented with a difficult ring was not an effective strategy. The requirement that the subject needed to pass through the rings without touching them added an additional level of difficulty to the task, and trained subjects to establish and modify the flight path as each situation required. The capacity for adjustment of the control plan during all stages of control is essential for real world applications. In these applications, goals will not be imposed by the system, but by the will of the user. Therefore, it is essential to allow the user to alter the flight path at any time to respond to a change in intent or as a reaction to an unexpected event.

The adapted multidimensional control system presented here is innovative in its incorporation of both force and displacement actuation to achieve fluid movement through the control, 3-dimensional space. The rotation of the helicopter was linearly tied to the control signal generated through learned modulation of ERD and ERS arising from movement of the right and left hands. Tying this control signal to the change in the helicopter's rotational angle at each simulation frame allowed subjects to use the most well established and easily learned control signal, generated from right versus left hand imagination, to rotate the helicopter with high resolution and set a course for the desired target. Displacements in the vertical and horizontal axes were controlled using force actuation. This is an appropriate choice since the summative nature of forces allows the subject to dynamically affect the direction of vertical and horizontal displacement, but once the desired movement is actuated, the forces applied will cause the helicopter to continue to drift in the desired direction. The subject may then focus attention on modulation of the high-resolution rotational control to ensure proper alignment in relation to the ring. This removes the requirement for a subject to be able to perform multiple motor imageries simultaneously, a task often complicated for the inexperienced user. At the same time, the arrangement preserves the potential for complex control through the use of simultaneous motor imaginations by the experienced user. The interplay between high and low-resolution movement, leveraged in the design of this system, is an essential component of the brain's ability to coordinate movement in three-dimensional space. When grasping a target with the hand, cognition is dedicated to small collections of motor units to coordinate the fine movement of the fingers. At the same time, larger groups of motor units are recruited to move the arm in the general desired direction. This intrinsic arrangement of the human nervous system is reflected in the design of this novel BCI.

### Conclusion

Three-dimensional control that is fast, accurate and continuous is a prerequisite for many of the useful applications envisioned for BCI. Here we present a novel system that allows users to navigate to a series of randomly positioned targets in 3D space. The system enables them to fly quickly and accurately through a series of rings in an unbroken path, characteristic of continuous control. Furthermore, utilizing BCI2000, a well-established software platform, we were able to successfully expand the limits of motor imagery based BCI into three dimensions. No BCI applications to date have allowed for this continuous, three-dimensional control along an unbroken path to multiple targets through the use of non-invasive EEG. By placing emphasis on the interplay between the methodologies used to train the user and the functionality of this novel system, the possibilities for non-invasive BCIs for potential applications to neuroprosthetics, rehabilitative medicine or other fields will continue to expand.

## Supporting Information

Table S1 **Customized spatial locations and frequency bins of subject control signals.** The left/right control signal components are positively weighted or negatively weighted if they are on the right or left side of the head, respectively. Therefore, their summation is a measure of the difference in ERS between the right and left motor cortex. The forward/backward components are summed with the same weight to quantify the overall degree of ERS. Subject 3's up/down control signal includes one negative component, FC4/12 Hz, which is likely located over or near the region of the motor cortex responsible for controlling tongue movements. Since the region below Cz usually encodes for leg movements, these components must be oppositely weighted to construct a viable tongue versus foot control method.
(DOCX)

Video S1 **Subject 1 acquires 3 rings continuously within 30 seconds.** The subject suppresses forward movement while lining up with the ring, and then accelerates forward to pass through it.
(MOV)

Video S2 **Subject 2 acquires 2 rings continuously within 30 seconds.** Similar to Video S1, this subject suppresses forward movement while lining up with the ring, and then accelerates forward to pass through it.
(MOV)

Video S3 **Subjects 2 exhibits control over backward movement to obtain the first ring in a series of continuous target acquisitions.** The attenuated, but effective, degree of backward movement in this video illustrates typical use for this control. The subject then switches to forward movement to pass through the second ring. Subjects reported that flying forward was more intuitive than in reverse because they were unable to see directly behind the helicopter.
(MOV)

Video S4 **Subject 3 acquires 3 rings continuously within 40 seconds.**
(MOV)
