# Supplementary material for: Continuous Three-Dimensional Control of a Virtual Helicopter Using a Motor Imagery Based Brain-Computer Interface
Source: PLoS One. 2011 Oct 26;6(10):e26322. doi: 10.1371/journal.pone.0026322 (PMC3202533; doi:10.1371/journal.pone.0026322)
Supplement: Table S1 — Customized spatial locations and frequency bins of subject control signals. The left/right control signal components are positively weighted or negatively weighted if they are on the right or left side of the head, respectively. Therefore, their summation is a measure of the difference in ERS between the right and left motor cortex. The forward/backward components are summed with the same weight to quantify the overall degree of ERS. Subject 3's up/down control signal includes one negative component, FC4/12 Hz, which is likely located over or near the region of the motor cortex responsible for controlling tongue movements. Since the region below Cz usually encodes for leg movements, these components must be oppositely weighted to construct a viable tongue versus foot control method. (DOCX) [file pone.0026322.s001.docx]

| **Table S1. Control Signals** | | | |
| --- | --- | --- | --- |
|  | Control Dimensions | | |
| Subject | Left/Right | Forward/Backward | Up/Down |
| S1 | C4/12,15,18 Hz (+) CP2/9,15 Hz (+) C3/12 Hz (-) | C4/12,15,18 Hz (-) CP2/9,15 Hz (-) C3/12 Hz (-) | Cz/12,24 Hz (+) FCz/12 Hz (+) |
| S2 | C4/15,18 Hz (+) C3/12 Hz (-) | C4/15,18 Hz (-) C3/12 Hz (-) | Cz/12 Hz (+) |
| S3 | C4/12 Hz (+)  C3/12 Hz (-) | C4/12 Hz (-)  C3/12 Hz (-) | Cz/12 Hz (+)  FC4/12 Hz (-) |
